# CD36 deficiency ameliorates drug-induced acute liver injury in mice

**DOI:** 10.1186/s10020-021-00325-z

**Published:** 2021-06-06

**Authors:** Chen Zhang, Xiao Shi, Zhongping Su, Chao Hu, Xianmin Mu, Jinshun Pan, Mengjing Li, Fengmeng Teng, Tao Ling, Ting Zhao, Che Xu, Guozhong Ji, Qiang You

**Affiliations:** 1 Department of Biotherapy, Medical Center for Digestive Diseases, Second Affiliated Hospital of Nanjing Medical University, Nanjing, 210011 China; 2 Affilated Hospital of Nanjing University of Chinese Medicine, Nanjing, 210029 China; 3 Affiliated Cancer Hospital and Institute of Guangzhou Medical University, Guangzhou, 510095 China; 4 Renji Hospital Affiliated to Shanghai Jiaotong University School of Medicine, Shanghai, China

**Keywords:** CD36, Acetaminophen, Hepatotoxicity, JNK, HMGB1, Sterile inflammation

## Abstract

**Background:**

Acetaminophen (APAP) overdose causes hepatotoxicity and even acute liver failure. Recent studies indicate that sterile inflammation and innate immune cells may play important roles in damage-induced hepatocytes regeneration and liver repair. The scavenger receptor CD36 has its crucial functions in sterile inflammation. However, the roles of CD36 in APAP induced acute liver injury remain unclear and warrant further investigation.

**Methods:**

WT C57BL/6 J and CD36^−/−^ mice were intraperitoneally injected with APAP (300 mg/kg) after fasting for 16 h. Liver injury was evaluated by serum alanine aminotransferase (ALT) level and liver tissue hematoxylin and eosin (H&E) staining. Liver inflammatory factor expression was determined by real-time polymerase chain reaction (PCR). The protein adducts forming from the metabolite of APAP and the metabolism enzyme cytochrome P450 2E1 (CYP2E1) levels were measured by Western blot. Liver infiltrating macrophages and neutrophils were characterized by flow cytometry. RNA sequencing and Western blot were used to evaluate the effect of damage-associated molecular patterns (DAMP) molecule high mobility group B1 (HMGB1) on WT and CD36^−/−^ macrophages. Moreover, PP2, a Src kinase inhibitor, blocking CD36 signaling, was applied in APAP model.

**Results:**

The expression of CD36 was increased in the liver of mice after APAP treatment. Compared with WT mice, APAP treated CD36^−/−^ mice show less liver injury. There was no significant difference in APAP protein adducts and CYP2E1 expression between these two strains. However, reduced pro-inflammatory factor mRNA expression and serum IL-1β level were observed in APAP treated CD36^−/−^ mice as well as infiltrating macrophages and neutrophils. Moreover, CD36 deficiency impaired the activation of c-Jun N-terminal kinase (JNK) caused by APAP. Interestingly, the lack of CD36 reduced the activation of extracellular regulated protein kinases (Erk) and v-akt murine thymoma viral oncogene homolog (Akt) induced by HMGB1. RNA transcription sequencing data indicated that HMGB1 has a different effect on WT and CD36^−/−^ macrophages. Furthermore, treatment with PP2 attenuated APAP induced mouse liver injury.

**Conclusion:**

Our data demonstrated that CD36 deficiency ameliorated APAP-induced acute liver injury and inflammatory responses by decreasing JNK activation. CD36 might serve as a new target to reduce acute liver injury.

## Introduction

Acetaminophen (APAP) overdose causes hepatotoxicity, and even acute liver failure, which involves a series of critical events including APAP metabolite protein adduct formation, mitochondrial dysfunction, oxidant stress, peroxynitrite formation and nuclear DNA fragmentation (McGill 2012). APAP hepatotoxicity results from the formation of a reactive metabolite, hepatic glutathione depletion, and protein binding, which correlates with liver injury (Jaeschke et al. 2012). The cell death is initiated by the formation of N-acetyl-p-benzoquinone imine (NAPQI), which is generated mainly by the cytochrome P450 enzymes CYP2E1 and 1A2 in mice and humans (Raucy et al. 1989; Zaher 1998). The detoxification of NAPQI by conjugation with glutathione (GSH) is impaired due to the limition of GSH after APAP overdose. Subsequently, NAPQI binds to proteins at sulfhydryl groups of cysteine which results in the impaired mitochondrial respiration and increased mitochondrial oxidant stress (Jaeschke 1990). Moreover, APAP overdose leads to the activation of the mitogen activated protein (MAP) kinase JNK and its translocation to the mitochondria, which triggers the mitochondrial membrane permeability transition (MPT). Consequently, the dysfunction of mitochondria causes nuclear DNA damage which contributes to cell necrosis. Recent studies indicate that sterile inflammation and innate immune cells may play important roles in damage-induced hepatocyte regeneration and liver repair (Woolbright and Jaeschke 2017). The cell necrosis leads to the release of damage-associated molecular patterns (DAMPs) including HMGB1 protein, DNA fragments, and heat shock proteins, which can be recognized by the receptors on monocytes and macrophages and results in their activation and inflammatory responses (Martin-Murphy et al. 2010; Jeannin et al. 2008; Schwabe et al. 2006).

CD36 is a member of the class B scavenger receptor family of cell surface proteins. It binds a variety of ligands including collagen, thrombospondin, erythrocytes parasitized with plasmodium falciparum, oxidized low density lipoprotein, native lipoproteins, oxidized phospholipids and long-chain fatty acids (Silverstein and Febbraio 2009). The ligands trigger signaling pathways consisting of immediate recruitment and activation of Src family kinases Fyn and Lyn, and MAPK JNK (Yang 2017). CD36 also assembles with Toll-like receptor 4 and 6 to promote sterile inflammation (Stewart 2010). It has been positioned as a central regulator of sterile inflammation by coordinating NACHT, LRR and PYD domains-containing protein 3 (NLRP3) inflammasome activation (Sheedy 2013). In the liver, CD36 was highlighted as a strong contributor to steatosis and hepatic injury in the context of proprotein convertase subtilisin/kexin type 9 (PCSK9) deficiency (Lebeau 2019). Circulating soluble CD36 could represent a novel marker of liver injury in subjects with altered glucose tolerance (Fernandez-Real 2009). Moreover, our previous data indicate that CD36 participates in Concanavalin A induced murine liver injury (Xu 2018). However, how and to what extent CD36 contributes to APAP induced hepatic injury has not been investigated.

In this study, we first examined the expression of CD36 in mouse liver with APAP-induced liver injury. Then, the extent of liver injury was compared between WT C57BL/6 J and CD36^−/−^ mice evaluated by serum ALT level, liver tissue H&E staining and Terminal deoxynucleotidyl transferase dUTP nick end labeling (TUNEL) assay. Moreover, inflammatory gene expression was determined by real-time PCR. RNA sequencing (RNA-seq) was adopted to evaluate the effect of HMGB1 on WT and CD36^−/−^ macrophages. Liver infiltrating macrophages and neutrophils were characterized by flow cytometry.

## Materials and methods

### Mice

Experiments were conducted with 6–8 weeks old male C57BL/6 J WT (Nanjing Biomedical Research Institute of Nanjing University) and CD36^−/−^ mice (on C57BL/6 J background, Jackson Laboratories). The mice were housed in a temperature-controlled environment with a 12 h light–dark cycle and were allowed free access to water and food. All animal procedures were approved by the Laboratory Animal Core Facility of Nanjing Medical University.

### Animal treatment and assessment of hepatotoxicity

The mouse model of APAP induced acute liver injury was performed as previously described (You 2013). The mice were fasted overnight for approximately 16 h prior to intraperitoneal (i.p.) injection of phosphate buffered solution (PBS) or APAP (300 mg/kg, dissolved in warm PBS, Sigma, USA) at 9 a.m. The samples were collected after 8 h at 5 p.m. and 24 h at 9 a.m. next day. Blood was collected by retro-orbital puncture. Sera samples were collected for measurement of serum ALT and aspartate aminotransferase (AST) levels using an automated chemistry analyzer (Beckman AU5800, BECKMAN COULTER, USA). Liver samples were obtained at various time points after APAP challenge and paraffin-embedded tissue sections were prepared and stained with H&E. The Src family kinase selective inhibitor PP2 (Selleck, China) was injected to the mice intraperitoneally (1.5 mg/kg, dissolved in ethanol) at half an hour prior to APAP-treatment.

### TUNEL assay

Necrotic cells were evaluated using the TUNEL assay kit (Roche Applied Science) according to the manufacturer’s instructions.

### Quantitative real-time PCR

Total RNA was extracted from liver tissues collected at indicated time after APAP injection using the Ultrapure RNA kit (Thermo Fisher Scientific, Invitrogen, MA, USA) and transcribed into cDNA using the reverse transcription kit (Thermo Fisher Scientific, Waltham, MA, USA). Subsequently, the resultant cDNA was amplified with the Maxima SYBR-Green/Rox q-PCR Master Mix 2X kit (Thermo Fisher Scientific, Waltham, MA, USA) using the Step One Plus Real-Time PCR System (Thermo Fisher Scientific). Primers used in the PCR-reactions were synthesized in Invitrogen (Shanghai, China) as followed: GAPDH (CAT CAC TGC CAC CCA GAA GAC TG, ATG CCA GTG AGC TTC CCG TTC AG); 18S ribosomal RNA (rRNA) (AGG GGA GAG CGG GTA AGA GA, GGA CAG GAC TAG GCG GAA CA); CD36 (GGA CAT TGA GAT TCT TTT CCT CTG, GCA AAG GCA TTG GCT GGA AGA AC); IL-1β (TGG ACC TTC CAG GAT GAG GAC A, GTT CAT CTC GGA GCC TGT AGT G); TNF-α (GGT GCC TAT GTC TCA GCC TCT T, GCC ATA GAA CTG ATG AGA GGG AG); IL-6 (TAC CAC TTC ACA AGT CGG AGG C, CTG CAA GTG CAT CAT CGT TGT TC); KC (ACT GCA CCC AAA CCG AAG TC, TGG GGA CAC CTT TTA GCA TCT T); MCP1 (TGT ACC ATG ACA CTC TGC AAC, CAA CGA TGA ATT GGC GTG GAA).

### Isolation of mouse liver non-parenchymal cells (NPCs)

Mouse primary liver cells were isolated following a previously established method (You et al. 2008). In brief, the mouse liver was perfused with Hank's balanced salt solution followed by a digestion buffer containing 0.2% collagenase (Type IV, Sigma). Single cell suspensions were filtered through a 100 µm cell strainer and centrifuged at 50 g for 3 min. Hepatocytes were in the pellet, and the cells in the supernatant were collected and further centrifuged in 35% percoll (Sigma, Germany) to obtain liver NPCs. Red blood cells were lysed with red blood cell lysing buffer. Liver NPCs were stained with fluorochrome-conjugated antibodies for multi-color fluorescent-activated cell sorting (FACS) analysis, including APC Cyanine7conjugated anti-mouse CD45 (clone 30F11, #130-105-506; Miltenyi Research Inc. San Diego, CA), APC conjugated anti-mouse F4/80 (clone BM8, #17-4801-82, eBioscience), PE-vio770 conjugate anti-mouse CD11b (clone M1/70, #25-0112-82, eBioscience), PE-conjugated anti-mouse Ly6G (clone RB6-8C5, #561084, BD Biosciences) and FITC-conjugated anti-mouse Ly6C (clone AL21, #553104, BD Biosciences, San Jose, CA). BD FACSCanto II flow cytometer was used and the software FlowJo (V10) was utilized to analyze data.

### Isolation of bone marrow derived macrophages (BMDM)

The mice were euthanized by carbon dioxide, and mouse tibiae and femurs were collected and flushed with ice-cold PBS through a 70 μm cell strainer. Bone marrow cells (5 × 10^6^/ml) were seeded on non-treated cell culture plates and cultured in the medium containing macrophage colony stimulating factor (10 ng/ml). The differentiated BMDM were harvested after culture for 7 days.

### RNA-sequencing and data analysis

BMDM were treated with PBS or HMGB1 (1 μg/ml) for 24 h and lysed in TRIzol reagent. Total RNA was isolated and used for RNA-sequencing analysis. cDNA library construction and sequencing were performed by Beijing Genomics Institute (BGI) using BGISEQ-500 platform. Bioinformatics Workflow including data filtering, mapping transcript prediction, differential gene expression analysis and GO and Kyoto Encyclopedia of Genes and Genomes (KEGG) pathway analysis were performed by the platform established at BGI.

### Histology analyses

The liver tissues were fixed in 10% formalin for 24 h followed by processing and paraffin embedding. Sections (4 μm) of paraffin-embedded tissues were stained with H&E. Immunohistochemical staining of CD36 was performed using recombinant anti-CD36 antibody (#ab133625, Abcam, Cambridge, MA, USA) at the dilution of 1:200. Stained tissue sections were evaluated under light-microscope (Olympus IX51, Japan).

### Western blotting

Total proteins were prepared from mouse liver tissues or cultured cell samples using STE buffer (100 mM Tris, 1 mM EDTA, 250 mM sucrose) or RIPA lysis buffer (50 mM Tris (pH 7.4), 150 mM NaCl, 1% NP-40, 0.5% sodium deoxycholate, 0.1% SDS) containing protease and phosphatase inhibitors cocktail (Roche Applied Science, Penzberg, Germany). The protein concentration was quantified by bicinchoninic acid (BCA) assay kit (Beyotime Biotechnology, Shanghai, China). Then, the lysate supernatants were heated in sodium dodecyl sulfate–polyacrylamide gelelectrophoresis (SDS-PAGE) sample-loading buffer (Beyotime Biotechnology). Protein extracts were separated on 8–15% SDS–polyacrylamide gels and transferred to the PVDF membrane (Bio-Rad Laboratories, Inc.). Then the protein was probed with specific primary antibodies followed by horseradish peroxidase conjugated antibody. The antibodies were used as follows: anti-CYP2E1 (#AB1252, Merck KGaA), anti-CD36 (#ab133625, Abcam), anti-phospho-Erk (#4370), anti-total Erk (#4695), anti-phospho-AKT (#9271), anti-total AKT (#4691), anti-phospho-JNK (#9251), anti-total JNK (#9258) and anti-β-Actin (#4970) (all from Cell Signaling Technology, Beverly, MA, USA). The antibody against the metabolite of APAP, NAPQI was kindly given by Professor Cynthia Ju (UTHealth, USA). Automatic digital gel image analysis system Tanon-4500 was used to capture chemiluminescence. The software Image J was utilized to calculate band density with β-actin as a control.

### ELISA

Mouse IL-1β ELISA Kit was purchased from Invitrogen (#BMS6002, Thermo Fisher Scientific, MA, USA). Mouse IL-6 ELISA Kit was obtained from Biolegend (#431304, San Diego, CA, USA). The measurement was performed according to manufacturer’s protocols.

### Statistical analysis

All data are represented as mean ± standard deviation. Statistical significance between groups were assessed by means of a two-tailed unpaired Student t test or ANOVA for comparison of two or multiple groups (GraphPad Prism, USA). Differences were considered significant when P < 0.05. All experiments were repeated at least three times.

## Results

### CD36 deficiency attenuates APAP-induced liver injury in mice

**Fig. 1:**
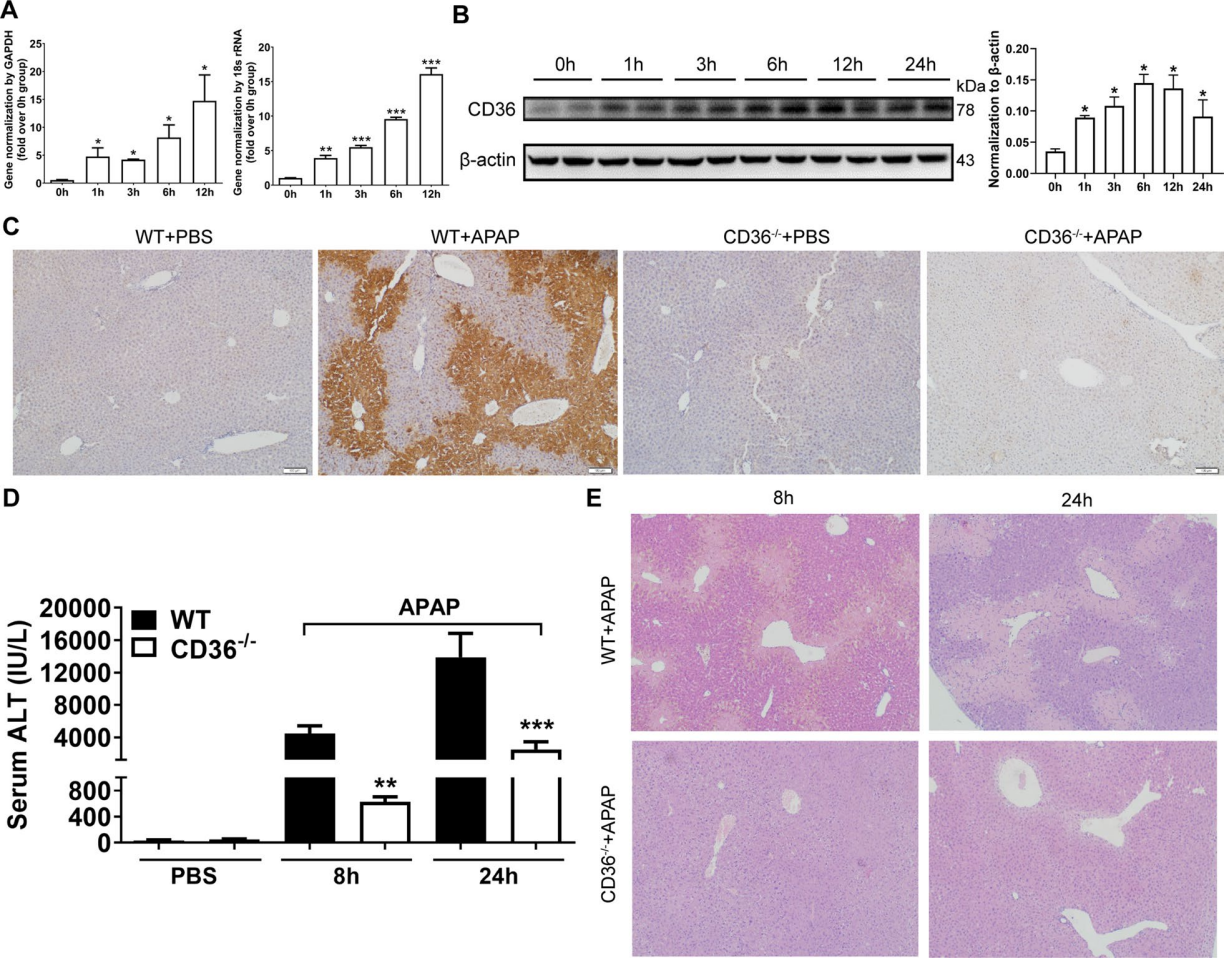
CD36 deficiency reduced APAP-induced murine liver injury. WT mice were starved for 16 h and i.p. injected with APAP at 300 mg/kg. The expression of CD36 in the liver at specified time point after APAP treatment was determined by **A** q-PCR and **B** Western blotting. *p < 0.05, compared with 0 h; **C** Immunohistochemical staining of CD36 in the liver sections of mice treated with PBS or APAP for 24 h. **D** Serum ALT and **E** liver H&E staining at 8 h and 24 h after APAP injection in mice. N = 6–8 mice per group. **p < 0.01, ***p < 0.001, compared with WT mice

In order to investigate the role of CD 36 in APAP-induced liver injury, the expression of CD36 level was determined in the livers from mice with APAP overdosage. As a result, the mRNA level of CD36 has increased four folds in WT mice 1 h after APAP treatment, and the peak occurred at 12 h (Fig. 1A). Correspondingly, the protein level of CD36 was gradually upregulated within 24 h after APAP overdose (Fig. 1B). Intriguingly, CD36 was mainly localized in the hepatic cells around the central vein (perivenular or zone 3) (Fig. 1C). Furthermore, the difference of liver injury was compared between WT and CD36^−/−^ mice with APAP treatment. As a result, serum ALT levels were significantly lower in CD36^−/−^ mice compared with those in WT mice at both 8 h and 24 h after APAP treatment (Fig. 1D). Accordingly, H&E staining shows that less necrotic areas were observed in the livers from CD36^−/−^ mice compared with that in WT mice (Fig. 1E).

### Unaltered APAP metabolism and reduced inflammatory responses in mice lacking of CD36

**Fig. 2:**
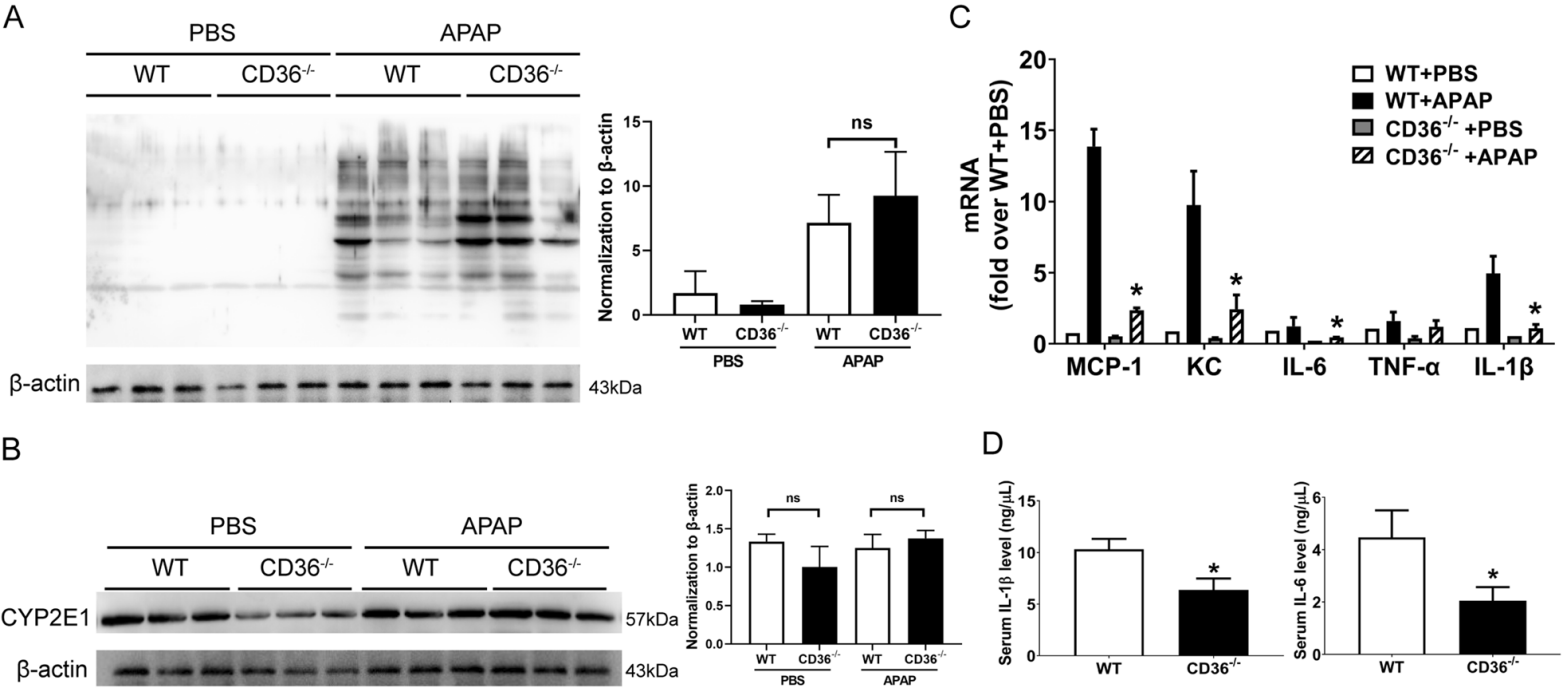
Lack of CD36 ameliorated liver inflammatory responses induced by APAP. The expression of **A** NAPQI-protein adducts and **B** CYP2E1 in the liver from WT and CD36^−/−^ mice treated with APAP for 6 h. **C** mRNA level of cytokines was determined by RT-qPCR in the liver of WT and CD36^−/−^ mice at 24 h after APAP treatment. **D** Serum IL-1β and IL-6 levels in WT and CD36^−/−^ mice at 24 h after APAP overdosage. *P < 0.05, compared with WT mice. N = 5 mice per group

APAP metabolite NAPQI covalently binds to cellular proteins and causes cell death (Jaeschke and Ramachandran 2018). Therefore, NAPQI-protein adducts were examined in WT and CD36^−/−^ mice with APAP overdose. As shown in Fig. 2A, there was no significant difference in the production of NAPQI-protein adducts between these two strains. Cytochrome P450 2E1 (CYP2E1) is the main P450 enzyme involved in oxidative APAP metabolism in the liver (Lee et al. 1996). However, the expression of CYP2E1 protein shows a similar level in WT and CD36^−/−^ mouse group (Fig. 2B). Furthermore, the expression level of inflammatory cytokines was investigated in the livers of WT and CD36^−/−^ mice. At 24 h after APAP injection, the mRNA levels of inflammatory factor MCP-1, KC, IL-6 and IL-1β were significantly decreased in CD36^−/−^ mice compared to those in WT mice (Fig. 2C). Accordingly, the levels of serum IL-1β and IL-6 were much less in the serum of APAP treated CD36^−/−^ mice than that in WT mice (Fig. 2D). These data suggest that CD36 deficiency leads to a reduction in APAP-induced hepatic inflammatory response.

### The lack of CD36 impairs the activation of JNK signaling in the liver

**Fig. 3:**
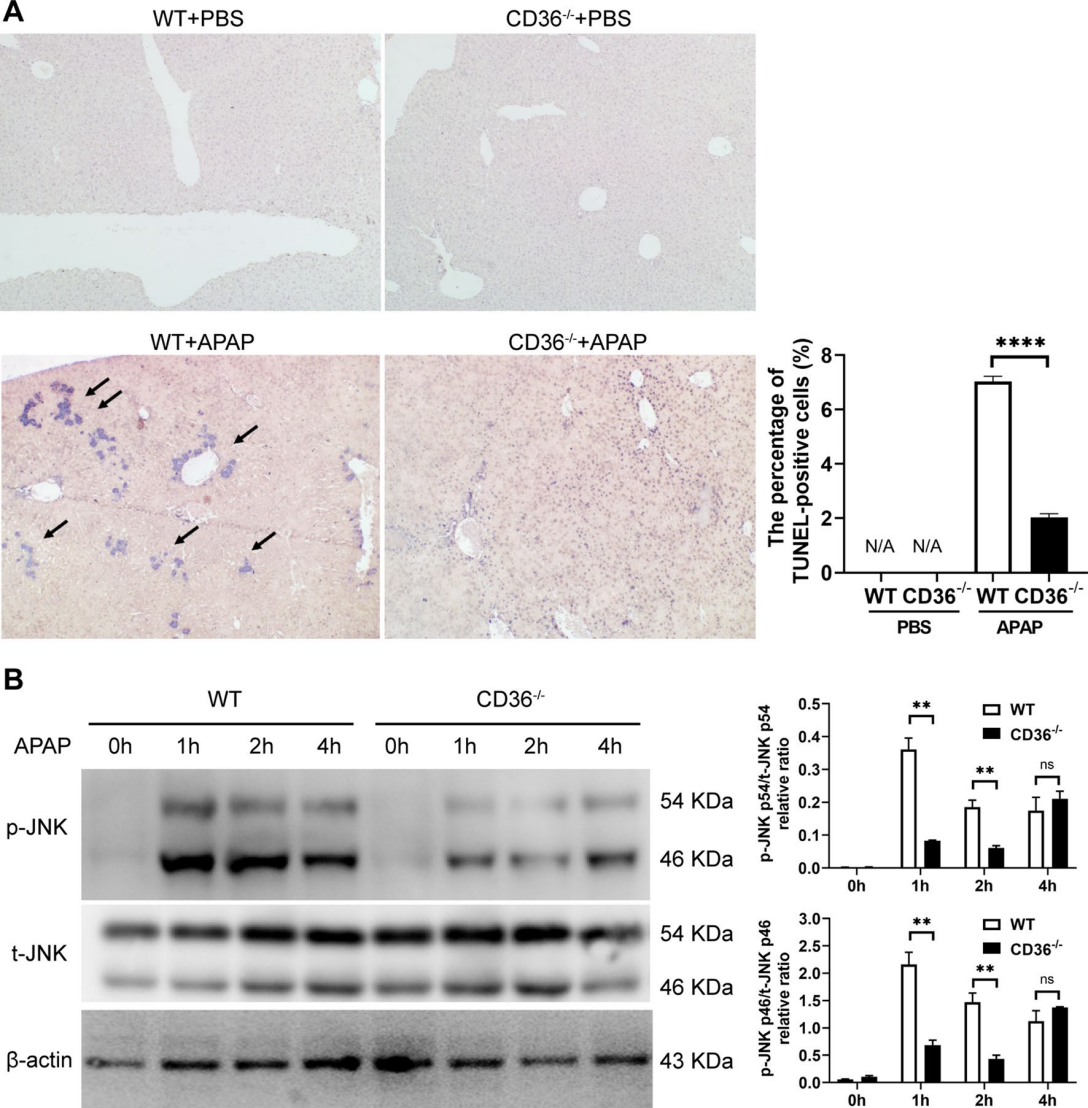
CD36 deficiency reduced APAP induced DNA fragmentation and activation of JNK. **A** Necrosis induced DNA fragmentation in mouse livers was measured by TUNEL assay at 24 h after APAP treatment. **B** The activation of JNK was determined by Western blot. **P < 0.01

DNA fragmentation is a characteristic feature of APAP-induced cell death. Then, the liver necrotic cells were examined by TUNEL assay. Consequently, the frequency of TUNEL^+^ cells were dramatically decreased in APAP-challenged liver lobes from CD36^−/−^ mice, as compared with WT mice (Fig. 3A). Intriguingly, a closer examination of staining in CD36^−/−^ mice shows positive staining, though this is now restricted to the nucleus of individual cells (Fig. 3A). It was recognized that JNK signaling plays a crucial role in APAP induced liver injury (Jaeschke and Ramachandran 2018). Therefore, the activation of the JNK signaling was further investigated at 24 h after APAP treatment. As a result, the level of phosphorylated JNK was increased upon APAP treatment. However, the absence of CD36 reduced the APAP-induced increment (Fig. 3B).

### CD36 deficiency reduces the infiltration of monocytes and neutrophils in the liver

**Fig.4:**
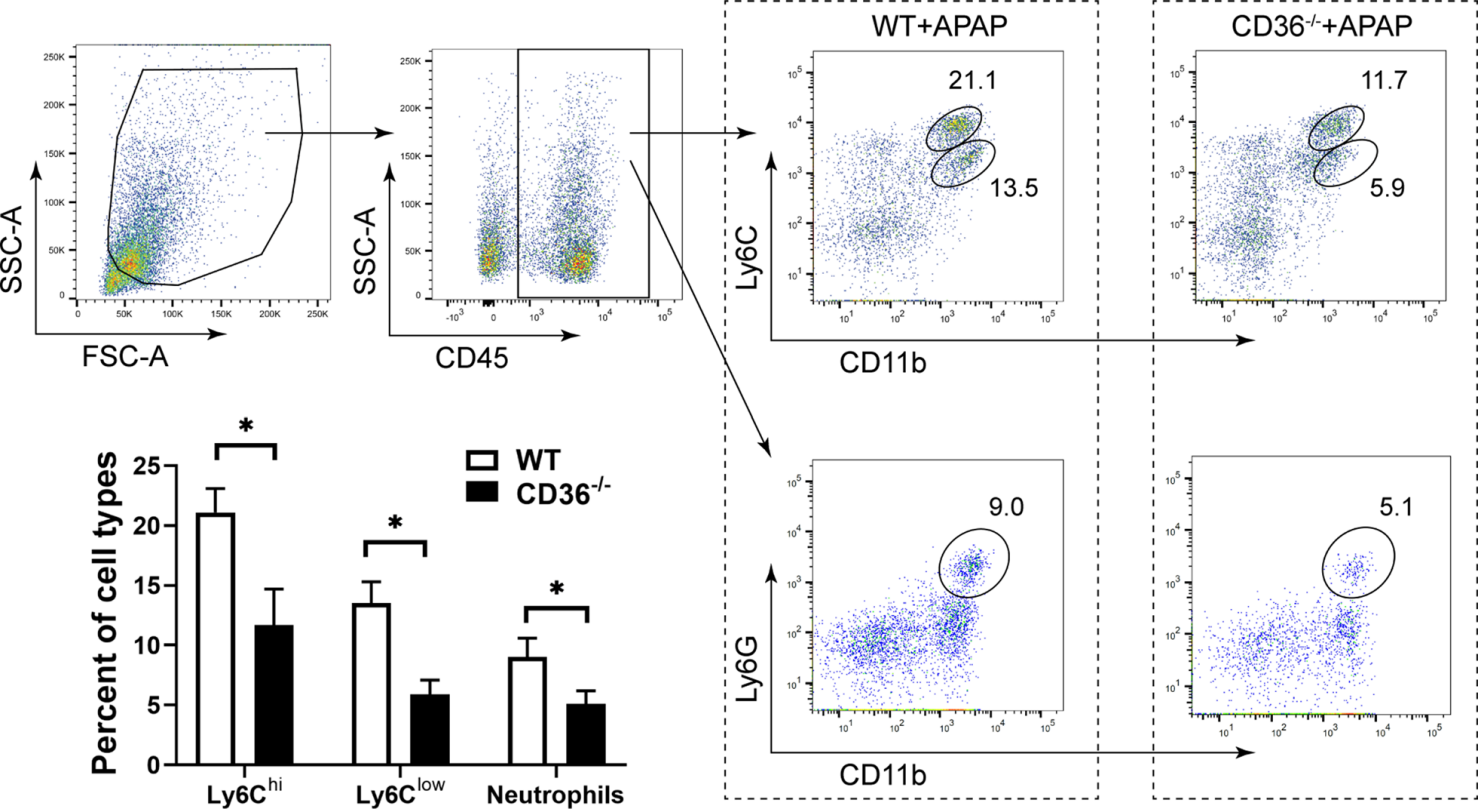
Infiltrating monocytes and neutrophils were deceased in CD36^−/−^ mice. LMNCs were purified from APAP treated mice and stained with CD11b, Ly6C and Ly6G antibodies. Infiltrating monocytes (CD11b^+^ Ly6C^+^) and neutrophils (CD11b^+^ Ly6G^+^) were analyzed by FACS. *P < 0.05. N = 6 mice per group

The extensive necrosis during APAP-induced liver injury results in a sterile inflammatory response and recruitment of inflammatory cells in the liver. Therefore, the infiltration of monocytes and neutrophils were determined in APAP-treated mice by fluorescence-activated cell sorting (FACS) analysis. CD45^+^ cells were gated to exclude endothelial cells, stellate cells and residue hepatocytes (Fig. 4). The expression of CD11b and Ly6C was determined to identify two subsets of infiltrating monocytes (IMs), CD11b^+^ Ly6C^hi^ (high expression) and CD11b^+^ Ly6C^low^ (low expression) cells, which have been identified in the injured liver and responsible for the inflammatory responses. Our data show that CD11b^+^ Ly6C^hi^ and CD11b^+^ Ly6C^low^ infiltrating monocytes were present in the liver from APAP treated WT mouse (Fig. 4). Moreover, these two subsets of IMs were much fewer in the liver from APAP treated CD36^−/−^mouse. Furthermore, the recruitment of phagocytes neutrophils (CD11b^+^ Ly6G^+^) were also observed in the liver from APAP treated mice, where the percentage of neutrophils was much lower in the CD36^−/−^ mouse liver.

### The activation of macrophages by HMGB1 is mediated by CD36

**Fig.5:**
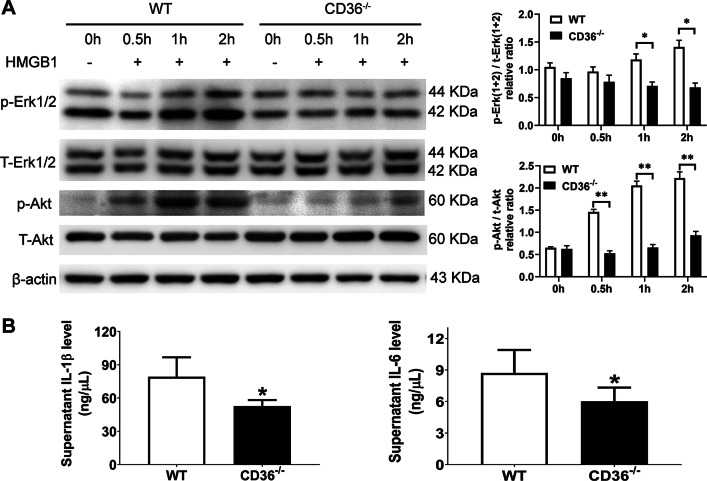
The effect of HMGB1 on BMDM from WT and CD36^−/−^ mice. BMDM were purified and treated with HMGB1 for various duration. The expression of p-Erk, total Erk, p-AKT and total AKT was examined by Western blot. The culture supernant IL-1β and IL-6 levels were measured from BMDM treated with HMGB1 for 48 h. *P < 0.05, **P < 0.01. N = 4 per group

Extensive cell necrosis causes the release of cell contents. HMGB1, one of the damage-associated molecular patterns (DAMPs), is found in the plasma after an APAP overdose (Antoine 2009). By binding cell-surface receptors on immune cells, HMGB1 activates intracellular signaling pathways that regulate immune cell function, including chemotaxis and cytokine production (Gaskell et al. 2018). Here, we sought to determine the effect of HMGB1 on the activation of macrophages from WT and CD36^−/−^ mouse. As a result, HMGB1 activated Erk and Akt signaling on bone marrow derived macrophages (BMDM) from WT mouse. Intriguingly, this effect was dramatically reduced in BMDM from CD36^−/−^ mouse (Fig. 5A). Accordingly, the culture supernatant IL-1β and IL-6 levels were significantly decreased in CD36^−/−^ BMDM (Fig. 5B).

### Comparison of the effect of HMGB1 on macrophages from WT and CD36^−/−^ mouse

**Fig.6:**
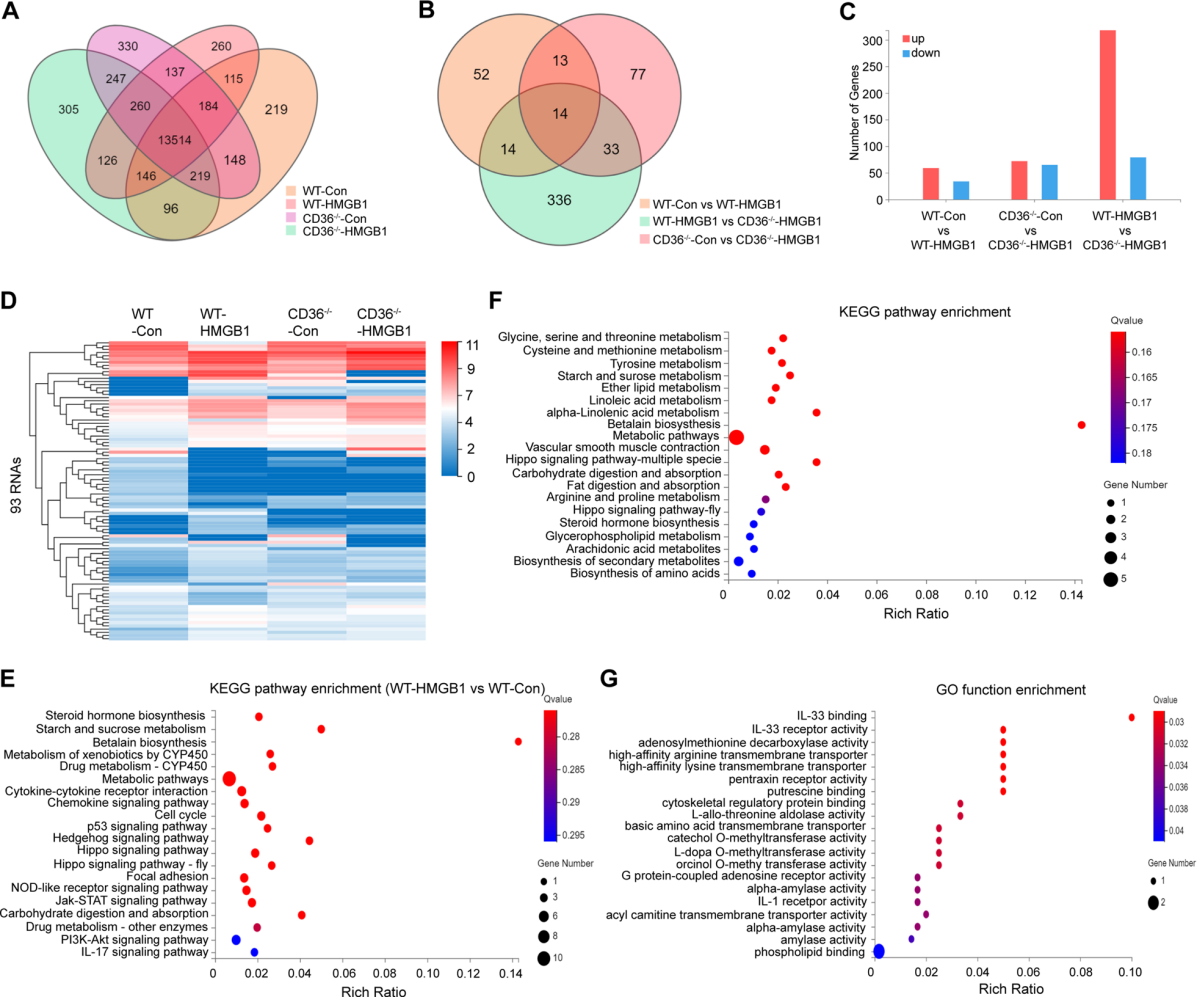
RNA-seq of control or HMGB1 treated BMDM from WT or CD36^−/−^ mice. **A** The VENN/UpSetR graph of RNA-seq for four groups of cells. **B** The different expression of genes between different comparison groups via VENN/UpSetR figure. **C** The significant differentially expressed genes (DEGs) detected were statistically plotted in three comparison. **D** The expression heat map of 93 genes in four groups of cells. **E** KEGG pathway enrichment bubble chart of the significant differentially expressed genes between WT-Con and WT-HMGB1 group. **F** KEGG and **G** GO function pathway enrichment bubble chart of RNAs significantly upregulated in HMGB1 treated BMDM from WT, not CD36^−/−^ mice. |log2FC|> = 1, FDR < 0.05

In order to comprehensively understand the effect of HMGB1 on macrophages from WT and CD36^−/−^ mouse, RNA-seq was conducted to further compare the gene expression between four groups of BMDM with different treatment, WT/Control (WT-Con), WT/HMGB1 treatment (WT-HMGB1), CD36^−/−^/Control (CD36^−/−^-Con) and CD36^−/−^/HMGB1 treatment (CD36^−/−^-HMGB1). As a result, the average RNA-seq depth was 22.11 million reads, with an average genome mapping rate of 96.59%, and total 16,338 genes were identified (Fig. 6A). There are 93 differentially expressed genes (59 upregulated and 34 downregulated) between WT-Con and WT-HMGB1 group, 136 differentially expressed genes between CD36^−/−^-Con and CD36^−/−^-HMGB1 group (72 upregulated and 65 downregulated), and 397 differentially expressed genes between WT-HMGB1 and CD36^−/−^-HMGB1 group (318 upregulated and 79 downregulated) (|log2FC|> = 1, FDR < 0.05) (Fig. 6B–D). KEGG pathway enrichment analysis of the 93 altered genes between WT-HMGB1 and WT-Con group show that metabolic pathways and chemokine signaling pathways are mainly involved (Fig. 6E). Furthermore, in 59 upregulated RNAs by HMGB1 treatment on WT BMDM, there are 29 RNAs which were not increased in HMGB1 treated CD36^−/−^ BMDM (Table 1). KEGG pathway analysis of these genes indicated an enrichment in metabolic pathways (Fig. 6F). Gene Ontology (GO)-function analysis showed an enrichment in the response to cytokine receptor and enzyme activity (Fig. 6G).

**Table 1:** The list of RNAs upregulated in HMGB1-treated WT BMDM, not in CD36^−/−^ BMDM

Gene symbol	log2 (WT + HMGB1/WT + Con)	log2 (CD36^−/−^ + HMGB1/CD36^−/−^ + Con)
Msmp	5.93	0.00
Rsc1a1	5.52	− 1.02
Amy2a2	5.13	0.67
Nptxr	3.32	− 0.58
Pakap	3.17	0.00
Phlda1	2.81	0.67
Tmem254a	2.76	− 8.26
Tomt	2.56	0.08
Xlr4c	2.54	− 1.87
Pla2g2d	2.08	0.48
Slc25a29	2.06	− 0.51
Slc12a8	1.86	0.58
Arl4d	1.74	− 0.50
C4a	1.74	− 0.29
Il1rl1/ST2/IL-33R	1.66	1.18
2210418O10Rik	1.34	− 8.16
Amd2	1.34	0.47
Fhod3	1.30	0.19
Zbed5	1.29	− 0.67
Gm4724	1.28	− 6.07
Adora2b	1.24	− 0.43
Rnase6	1.18	0.28
Tha1	1.16	− 0.33
Ajuba	1.13	0.00
Map1b	1.12	0.12
Gm46339	1.07	0.03
Mdk	1.07	− 0.56
Xlr4a	1.03	− 0.23
Zfp791	1.00	0.26

### The Src family kinase selective inhibitor PP2 ameliorated APAP induced acute liver injury

**Fig.7:**
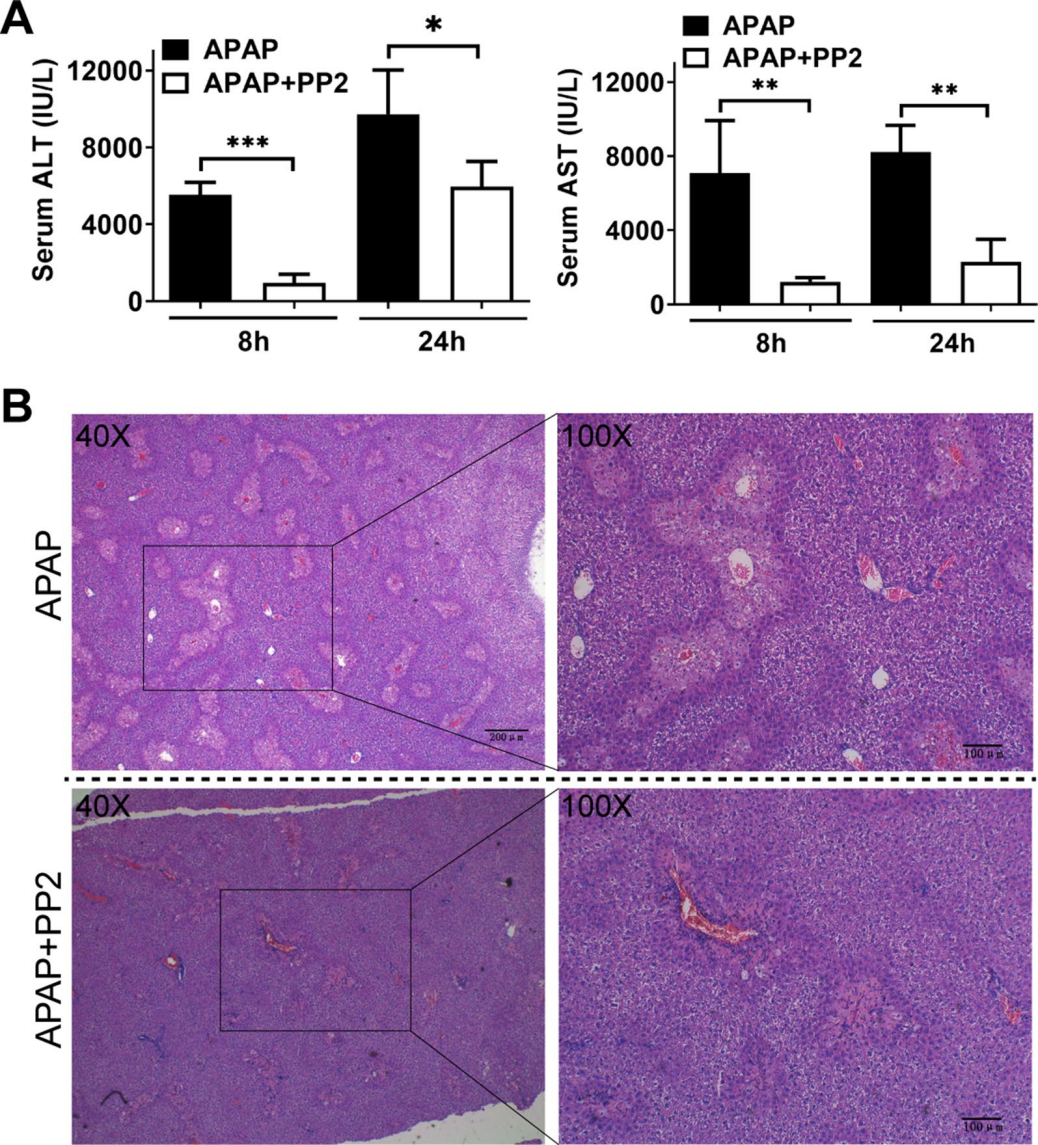
Treatment with PP2 attenuated APAP induced liver injury. **A** Male C57 mice were starved for 16 h and i.p. injected with/without PP2 (1.5 mg/kg) prior to APAP administration (300 mg/kg). Serum was collected after 8 h and 24 h for ALT and AST measurement. **B** H&E staining in liver section from the mice at 24 h after APAP injection. *P < 0.05, **P < 0.01, ***P < 0.001. N = 6 mice per group

It has been shown that the Src kinase inhibitor blocked the CD36 dependent activation (Stewart 2010; Hao 2020). Therefore, we treated WT C57BL/6 J mice with PP2 to elucidate the role of CD36 signaling in APAP–induced liver injury. Consequently, administration of PP2 significantly attenuated serum ALT and AST levels after APAP-treatment (Fig. 7A). Accordingly, the necrotic area was partially reduced after PP2 treatment (Fig. 7B).

## Discussion

APAP induced liver injury occurs by a complex sequence of events including CYP metabolism to a reactive metabolite, loss of glutathione with an increased formation of reactive oxygen and nitrogen species, increased oxidative stress, mitochondrial permeability transition, and loss of adenosine-triphosphate (ATP), which eventually leads to necrosis (Hinson et al. 2010). There appear to be a number of inflammatory mediators such as certain cytokines and chemokines that can directly promote intracellular injury or inhibit cell death and promote hepatocyte proliferation (Jaeschke et al. 2012). CD36, a scavenger receptor expressed in multiple cell types, mediates lipid uptake, immunological recognition, inflammation, molecular adhesion and apoptosis (Wang and Li 2019). In the present study, we sought to investigate the function of CD36 in acute liver injury.

APAP-induced hepatic necrosis mainly occurs in the centrilobular region (Yang 2012). Correspondingly, our data show that CD36 expression is increased after APAP overdosage and mainly localized in centrilobular area which indicates its potential relevance to liver injury. Moreover, CD36 deficiency ameliorated acute liver injury indicated by serum ALT level, H&E staining and TUNEL assay. In order to probe the mechanism, we first evaluate the drug metabolism in mice. CYP2E1 is widely accepted as the major isoform responsible for the bioactivation of APAP (Chen et al. 2008a). However, there is no difference of its level observed between WT and CD36^−/−^ mouse. Accordingly, the expression of NAPQI protein adducts was also similar. These data indicate that CD36 deficiency does not alter APAP metabolism in the liver.

CD36 is recognized as a fatty acid receptor which can bind lipid-related ligands, including long-chain free fatty acids, oxidative low-density lipoprotein and oxidized phospholipids (Zhao 2019). Therefore, CD36 deficiency may lead to lipid accumulation which can cause reactive oxygen species generation. Actually, lipid peroxidation is thought as a mechanism of cell death during APAP hepatotoxicity (Yoshioka 2017). However, it has been believed that lipid peroxidation is not a relevant mechanism of cell death but can be a marker of reactive oxygen species (ROS) formation, the electron transport chain of mitochondria is the main source of the oxidant stress (Jaeschke and Ramachandran 2018).

Protein binding and mitochondrial damage are central in the toxicity of APAP. The initial reactive oxygen species formation leads to activation of JNK. The translocation of active JNK into mitochondria exacerbates the oxidative stress and cell injury (McGill and Jaeschke 2013). Moreover, a JNK inhibitor can protect against injury (Saito et al. 2010). Therefore, the activation of JNK was evaluated in the livers from WT and CD36^−/−^ mice with APAP overdose. Encouragingly, the expression of pJNK was significantly lower in CD36^−/−^ mice. It has been reported that JNK activation was mediated by CD36 in various types of cells including macrophages (Rahaman 2006), platelets (Chen et al. 2008b) and adipocyte (Hao 2020). Thus, lack of CD36 seems to be preventing JNK activation, which is mechanistically upstream of mitochondrial dysfunction and hepatocyte necrosis induced by APAP. Although the extent of DNA fragmentation was lower in CD36^−/−^ mice than that in WT mice as measured by TUNEL assay, there is still positive staining in CD36^−/−^ mice which is now restricted to the nucleus of individual cells. This could be indicative of selective apoptosis in hepatocytes as a result of CD36 deficiency. Similar change in mechanism of cell death due to block of upstream APAP-induced pathways has been described recently. Treatment with the antioxidant Mito-tempo (MT), a mitochondria-targeted superoxide dismutase mimetic, may switch the mode of cell death to secondary apoptosis (Du 2019).

CD36 has been also positioned as a central regulator of sterile inflammation by coordinating NLRP3 inflammasome activation (Sheedy 2013). Cell death leads to the release of DAMPs including DNA fragment, HMGB1, and heat shock proteins, which result in sterile inflammation (Chen and Nunez 2010). The resultant inflammatory cytokines are able to modulate intracellular events within hepatocytes, thereby altering toxicity. Our data show that the expression of inflammatory gene MCP-1, KC, IL-6 and IL-1β was significantly reduced in CD36^−/−^ mice compared with that in WT mice. IL-6 is important in hepatocyte regeneration following APAP toxicity in the mouse (James et al. 2003). It is still controversial whether IL-1α and IL-1β are critical mediators in APAP hepatotoxicity (Jaeschke et al. 2012). It was reported that IL-1 receptor-deficient mice are completely protected from APAP induced liver injury (Chen 2007). However, another study shows that the liver damage and the hepatic neutrophilic inflammation were not attenuated in IL-1-receptor-1 deficient mice compared to wild-type animals (Williams et al. 2010). Our previous data revealed that mice deficient in the C–C chemokine receptor 2 (CCR2), the receptor for MCP-1, experienced reduced infiltrating macrophage accumulation during APAP hepatotoxicity and consequently a substantial delay in tissue repair (You 2013). We also demonstrated that IMs can be divided into two subsets based on their differential expression of Ly6C, where Ly6C (low) IMs exhibit an anti-inflammatory and tissue-protective phenotype; in contrast, Ly6C (hi) IMs exhibit a proinflammatory, tissue-damaging phenotype (Wang 2014). In the present study, both liver IM subsets were reduced in CD36^−/−^ mice compared with those in WT mice as well as neutrophils. It was reported that APAP induced hepatocyte death is amplified by liver neutrophil infiltration (Marques 2012). Mitochondrial DNA released by damaged hepatocytes activates neutrophils through binding of Toll-like receptor 9, further aggravating liver injury (He 2017; Imaeda 2009). However, it is also believed that neutrophils does not contribute to the liver injury after an APAP overdose (Jaeschke et al. 2012). Nevertheless, neutrophils may play an important role in liver repair by promoting phenotypic transformation of pro-inflammatory Ly6C (hi) macrophages to pro-repair Ly6C (low) macrophages (Yang 2019). Here, our data demonstrate that neutrophil recruitment was also reduced in CD36^−/−^ mice after APAP challenge compared with that in WT mice.

HMGB1 has been found in the plasma after an APAP overdose (Antoine 2009), and is an important mediator of the inflammatory response, as evidenced by that HMGB1 antibodies attenuated TNF-α, MCP-1, IL-6 formation (Jaeschke et al. 2012). More importantly, an HMGB1 neutralizing chimeric antibody attenuates APAP-induced liver injury and postinjury inflammation in mice (Lundback 2016). Therefore, we sought to determine the effect of HMGB1 on the activation of macrophages from WT and CD36^−/−^ mice. Our data demonstrate that HMGB1 activated Erk and Akt signaling in BMDM from WT mouse, not from CD36^−/−^ mice, which indicates that the effect of HMGB1 on macrophage may be through CD36 molecule. Subsequently, RNA-seq was employed to further comprehensively profile the function of CD36. It seems that the altered genes induced by HMGB1 are mainly involved in metabolic pathways and chemokine signaling pathways. Moreover, the absence of CD36 reduced the upregulation of the genes by HMGB1 which are related to metabolic pathways. Subsequently, we examined the direct interaction between CD36 and HMGB1 protein. However, there was no strong binding ability detectable (data not shown). How HMGB1 functions through CD36 molecule warrants further investigation. PP2, the CD36 signaling inhibitor, was further administered in the mouse model of acute liver injury. Notably, our data demonstrated that treatment with PP2 attenuated APAP induced liver damage.

In conclusion, we demonstrate that the scavenger receptor CD36 plays an important role in APAP-induced liver injury by impacting on the activation of JNK. Moreover, CD36 participates in the process of HMGB1-mediated inflammatory response. Our findings suggest a potential use of PP2 to treat APAP induced liver injury. CD36 may therefore serve as a target for therapeutic intervention in acute liver failure.

## Data Availability

The data that support the findings of this study are available from the corresponding author upon reasonable request.

## Abbreviations

Akt: V-akt murine thymoma viral oncogene homolog
ALT: Alanine aminotransferase
APAP: Acetaminophen
AST: Aspartate aminotransferase
ATP: Adenosine-triphosphate
BCA: Bicinchoninic acid
BMDM: Bone marrow derived macrophages
Erk: Extracellular regulated protein kinases
FACS: Fluorescence-activated cell sorting
H&E: Hematoxylin–eosin staining
HMGB1: High mobility group proteins B1
IMs: Infiltrating monocytes
i.p.: Intraperitoneal
M-CSF: Macrophage colony stimulating factor
NLRP3: NACHT, LRR and PYD domains-containing protein 3
JNK: C-Jun N-terminal kinase
KCs: Kupffer cells
MCP-1: Macrophage chemotactic protein-1
NAPQI: N-acetyl-p-benzoquinone imine
NPCs: Nonparenchymal cells
PCR: Polymerase chain reaction
PBS: Phosphate buffered solution
pJNK: Phosphorylated JNK
ROS: Reactive oxygen species
RNA-seq: RNA sequencing
SDS-PAGE: Sodium dodecyl sulfate–polyacrylamide gelelectrophoresis
TUNEL: Terminal deoxynucleotidyl transferase dUTP nick end labeling
